# Genetic analyses and molecular associations of *FSHR* and *GH* genes for semen traits in Egyptian buffalo

**DOI:** 10.1186/s12917-025-04866-x

**Published:** 2025-07-04

**Authors:** Abdelfatah R. Zaghloul, Maher H. Khalil, Mahmoud M. Iraqi, Amin M. S. Amin, Ibrahim Abousoliman, Ayman G. EL Nagar

**Affiliations:** 1 Department of Animal Production, Faculty of Agriculture, Benha University, Kalyoubia Governorate, Moshtohor Tukh, 13736 Egypt; 2 Animal Production Research Institute, Agricultural Research Center, Ministry of Agriculture, Dokki, Egypt; 3 Animal and Poultry Breeding Department, Desert Research Center (DRC), Mataryia Cairo, 11753 Egypt

**Keywords:** Egyptian buffalo, Semen traits, Genetic and phenotypic trends, *FSHR* gene, *GH* gene, PCR–RFLP

## Abstract

**Background & objectives:**

The reproductive efficiency of buffalo bulls is crucial for genetic improvement, herd fertility, and overall productivity. Identifying genetic markers linked to semen traits can thus enhance breeding programs and optimize artificial insemination strategies. The objectives of this study were to estimate variance components and heritability. estimating the breeding values (EBVs), plotting the genetic and phenotypic trends and detection of the molecular genetic associations of *FSHR* and *GH* genes using PCR–RFLP with semen traits comprising ejaculate volume (EV), sperms motility (SM), live sperms (LS), abnormal sperms (AS) and sperms concentration (SC) in Egyptian buffalo.

**Methods:**

Data of 5178 semen ejaculates were collected from 2013 to 2022 from 111 bulls, progeny of 34 sires and 92 dams in two experimental herds. For molecular genetic analysis, a total of 86 buffalo bulls were used to characterize *FSHR* and *GH* genes.

**Results:**

The heritabilities estimates for semen traits were low and moderate, being 0.17, 0.28, 0.27, 0.27 and 0.23 for EV, SM, LS, AS and SC, respectively. Wide ranges of the EBVs were observed, being -0.69 to 1.27 ml for EV, -18.19 to 11.59% for SM, -19.31 to 9.15% for LS, -2.05 to 6.41% for AS and -0.39 to 0.54 × 10^9^ sperms/*ml* for SC. The averages of EBV throughout different years of semen collection were ranged from -0.26 to 0.43 ml for EV, -9.73 and 3.32% for SM, -9.99 and 3.45% for LS, -0.65 to 0.53% for AS and -0.19 to 0.13 × 10^9^ sperms per ml for SC. The phenotypic trends plotted throughout the experimental period increased for all semen traits except for EV. The GLSM of the semen phenotypic values were ranged from 3.09 to 3.86 ml for EV, 61.55 and 66.53% for SM, 60.91 and 65.12% for LS, 4.34 to 9.28% for AS and 0.73 to 1.33 × 10^9^ sperms per ml for SC.The differences in generalized least square means among GG, GC and CC genotypes of *FSHR* gene for semen traits were significantly in favor of GG genotype relative to GC and CC genotypes (P < 0.01). Two genotypes of TC and CC were detected for *GH* gene and the molecular genetic associations were significantly in favor of CC genotype relative to TC genotype (P < 0.01).

**Conclusion:**

Enhancing management and feeding practices, the implementation and widespread use of artificial insemination as well as employing precise estimations of predicted breeding values in genetic improvement programs, should effectively enhance the semen traits of Egyptian buffalo bulls. *FSHR* and *GH* genes could be used as potential candidate genes for marker-assisted selection to improve semen traits in buffalo bulls.

## Introduction

In evaluating the reproductive efficiency in breeding programs of Egyptian buffalo, heritability and predicted breeding values are needed to investigate the genetic and phenotypic trends for semen traits and consequently to evaluate accurately the reproduction plans. The BLUPF90 software [1] is widely recognized as the global standard methodology for estimating the breeding values (EBV) for economic traits in buffalo. As stated by some Egyptian buffalo studies [2–4]**,** heritability estimates for semen traits were mostly low or somewhat moderate and ranged from 0.08 to 0.40 for ejaculate volume, 0.06 to 0.42 for sperms motility, 0.09 to 0.41 for live sperms percentage and 0.46 to 0.49 for sperms concentration. The ranges in breeding values for semen traits in buffalo are high, being −0.45 to 3.32 ml for ejaculate volume, −4% to 52% for sperms motility, −5.8 to 8.1% for live sperms and 799 to 1959 × 10^9^ for sperms concentration [2, 5]. Additionally, the studies concerning the genetic and phenotypic trends for semen traits in buffalo are limited. However, Kumar et al. [5] reported that the genetic and phenotypic trends were positive and showing favorable increase in ejaculate volume and sperms motility in the Indian buffalo bulls**.**

In the last decade, some molecular buffalo studies are providing the molecular markers to be used in marker assisted selection programs (MAS) and consequently to improve the selection response of semen traits in Egyptian buffalo [6–9], in Murrah buffalo [10, 11], and in Chinese buffalo [12]. In this concept, the Egyptian buffalo studies reported that *FSHR* gene is considered as an important candidate gene for reproduction, fertility and semen traits [13–16]. The *GH* gene has numerous biological roles, including those related to milk production, water and electrolyte balance, and reproduction and therefore *GH* gene may be utilized as a potential gene for the genetic improvement of buffalo [17]. Also, Darwish et al. [8] have shown that there were polymorphic associations between *GH* gene and semen quality traits in dairy bulls and buffalo. Therefore, the objectives of the current study were: 1) Estimating heritability and permanent environmental effects for some semen traits including ejaculate volume (EV), sperms motility (SM), live sperms (LS), abnormal sperms (AS) and sperms concentration (SC) in Egyptian buffalo, 2) Estimating the breeding values (EBVs) and plotting the genetic and phenotypic trends for semen traits, and 3) Detecting the molecular genetic associations between *FSHR* and *GH* genotypes and semen traits in Egyptian buffalo using PCR–RFLP.

## Materials and methods

### Management and feeding system

The buffalo bulls were raised in two herds of the International Livestock Management Training Center at Sakha (IMTC) and Mahalet Mousa (MM), Kafr El-Sheikh Governorate, belonging to Animal Production Research Institute (APRI), Agriculture Research Center, Ministry of Agriculture, Egypt. All the bulls were free of any clinical diseases with healthy appearances. According to APRI rules, the management and feeding regimes offered for the buffalo bulls were described and explained previously by Salem et al. [4].

## Semen collection and evaluation

A total of 5178 semen ejaculates were collected from 111 Egyptian buffalo bulls (weighing 350–400 kg in live body weight) produced from 34 sires and 92 dams during 10 years from 2013 to 2022. The bulls aged 18 to 24 months with scrotal circumference of more than 19 cm were used for insemination. Semen was collected individually from each bull at 8 AM using an artificial vagina (IMV, France) set up at optimal conditions to induce good ejaculatory thrust. At the time of semen collection, another buffalo bull was used as a teaser for sexual preparation. Ejaculates were obtained from each bull once a week at early morning (8:00 AM) throughout four consecutive weeks during four seasons of the year. The ejaculate volume was measured directly after ejaculation using a graduated glass tube. The sperms motility%, live sperms% and sperms concentration (10^9^ sperms per *ml*) were assessed according to Vale et al. [18]. Abnormal sperms% was evaluated according to the procedure adopted by Barbas and Mascarenhas [19].

### Data structure and animal model used in analyses

All the known relationships among animals were considered in analyses of semen traits and the pedigree file comprising a total of 10,802 animals with or without records born during the period from 1970 to 2023 and nine overlapped generations were used. The number of buffalo animals and records belonging to the two studied herds used in data analyses for semen traits are shown in Table 1.

**Table 1:** The number of buffalo animals and records belonging to the two herds used in data analyses of semen traits

Item	IMTC herd	MM herd	Both herds
Number of bulls with records	7	104	111
Number of sires with progeny and records	5	29	34
Number of dams with progeny and records	5	87	92
Total number of animals (bulls, sires and dams)	17	220	237
Total number of semen records	566	4612	5178

IMTC = International Livestock Management Training Center herd at Sakha and MM = Mahalet Mousa herd

The following repeatability single-trait animal model was fitted to the semen phenotypic data:

$$y=Xb+Z_au_a+Z_pu_p+e$$

where y = the vector of observed semen trait (EV, SM, LS, AS or SC) for the buffalo bull; b = the vector of fixed effects of herd (two levels; IMTC and MM), year-season of semen collection (38 levels) and age of the bull at semen collection (ten levels; 21–30, 31–40, 41–50, 51–60, 61–70, 71–80, 81–95, 96–110, 111–125 and ≥ 126 month of age); u_a_ = the vector of random additive genetic effect of the bulls; u_p_ = the vector of random non-additive permanent environmental effects of the buffalo bulls; X, Z_a_ and Z_p_ = the incidence matrices relating records to the fixed effects, additive genetic effects and permanent environment effects, respectively; e = the vector of random error. Heritabilities (h^2^) for semen traits were computed using the TM software of Bayesian Gibbs Sampling Algorithm as reported by Legarra et al. [20]: ${h}^{2}=\frac{{{\sigma }^{2}}_{a}}{{{\sigma }^{2}}_{a}+{{\sigma }^{2}}_{P}+{{\sigma }^{2}}_{e}} ,$ where ${{\sigma }^{2}}_{a}$ = the additive genetic variance of semen traits, ${{\sigma }^{2}}_{P}$ the permanent environmental variance and ${{\sigma }^{2}}_{e}$ = the residual variance. Then after, to find the solutions of the non-genetic effects and their error variance–covariance matrix, the PEST program [21] was utilized to solve the corresponding mixed model equations using the variances derived from Gibbs sampling obtaining the generalized least-square means (GLSM) of semen traits.

### Estimating the breeding values (EBVs) and plotting the genetic and phenotypic trends

Using the BLUPF90 software package [1], the estimated breeding values (EBVs), predicted error variance (PEV), and accuracies of predictions (r_A_) for EV, SM, LS, AS and SC were estimated. The same repeatability single-trait animal model previously described was used to estimate the EBV values, but replacing the year-season combination by the year of semen collection (10 years from 2013 to 2022). The accuracy of predicted breeding values (r_A_) was computed using the methodology mentioned by Meyer [22]. The phenotypic trends were plotted by regressing the GLSM of the phenotypic values of EV, SM, LV, AS and SC on year of semen collection. The breeding values for semen traits of 237 animals with and without records were estimated by BLUPF90 software [1]. The EBVs for bulls with records and parents without records were used in plotting the genetic trends by regressing the annual mean of the breeding values of EV, SM, LV, AS and SC on year of semen collection. Regression coefficients, intercept, R^2^ and P-values were estimated by SAS PROC REG using SAS software (SAS, Version 9.4. SAS Institute Inc., Cary, North Carolina, USA). Graph trends were obtained using Microsoft Excel 2016.

### Animals, blood sampling, DNA extraction, PCR amplification and genotyping by PCR–RFLP

A total of 86 buffalo bulls from Mahalet Mousa herd were used to assess the effect of the genetic polymorphisms of *FSHR* and *GH* genes on semen traits in Egyptian buffalo. All the available relationships among the animals were considered as A^−1^ matrix in molecular genetic data analyses. Blood samples were collected from 86 buffalo bulls belonging to Mahalet Mousa herd and a total of 72 animals (about 84% of the total blood samples) were successfully genotyped using PCR–RFLP. DNA samples were extracted from whole blood according to the extraction procedure illustrated by EL Nagar et al. [9] and PCR amplification and PCR–RFLP technique using *AluI* restriction enzyme for *FSHR* and *GH* genes were previously illustrated by Zaghloul et al. [23]. The primers used in the amplification process were given in Table 2. The molecular analyses including PCR amplification, PCR–RFLP and Gel electrophoresis have been performed in the Molecular Genetics Laboratory, Department of Animal and Poultry Breeding, Desert Research Center, Ministry of Agriculture, Egypt.

**Table 2:** Primers sequence and Annealing temperatures for *FSHR* and *GH* genes

Gene	Chr*	Primer sequences	PCR Product size (bp)	Annealing temp (ºC per time, s)
*FSHR*	12	F: CTGCCTCCCTCAAGGTGCCCCTC R: AGTTCTTGGCTAAATGTCTTAGGGGG	306	60/30
*GH*	3	F: GCTGCTCCTGAGGGCCCTTC R: CATGACCCTCAGGTACGTCTCCG	211	62/60

^*^Chr = Chromosome number

### Model for detecting polymorphic associations

The molecular genetic associations of different genotypes of *FSHR* and *GH* genes with semen traits were assessed using the previous repeatability single-trait animal model after adding the genotypes effect of *FSHR* gene (3 levels, GG, GC and CC) or *GH* gene (2 levels, TC and CC) as fixed effects. The GLSM of semen traits for different genotypes of *FSHR* and *GH* genes were estimated using PEST software [21].

## Results

### Descriptive statistics, heritabilities and permanent environmental effects

The generalized least square means (GLSM), standard deviations (SD), minimum and maximum values, coefficients of variation (CV %), heritability estimates and permanent environmental effects for semen traits are shown in Table (3). The GLSM for EV, SM, LS, AS and SC were 3.7 ml, 63.8%, 62.9%, 5.06% and 0.83 × 10^9^ sperms per *ml*, respectively. The ranges between minimum and maximum values for semen traits in Egyptian buffalo were high, being 1.0 to 10.5 ml for EV, 10 to 95% for SM, 10 to 88% for LS, 3 to 44% for AS and 0.2 to 3.8 × 10^9^ sperms per *ml* for SC (Table 3). The coefficients of variation (CV%) for semen traits were moderate or high, being 46% for EV, 28% for SM, 27% for LS, 55% for AS and 50% for SC (Table 3). The heritability estimates for semen traits were moderate, being 0.17, 0.28, 0.27, 0.27 and 0.23 for EV, SM, LS, AS and SC, respectively (Table 3). The proportions of permanent environmental effects (*p*^*2*^) were moderate for EV, SM, LS, AS and SC, being 0.16, 0.37, 0.35, 0.43 and 0.46, respectively (Table 3).

**Table 3:** Descriptive statistics, heritability estimates (h^2^), proportions of permanent environmental effects (*p*^*2*^) and random error effects (e^2^) for semen traits of Egyptian buffalo

Item	EV (*ml*)	SM (%)	LS (%)	AS (%)	SC (10^9^ sperms per *ml*)
Descriptive statistics^+^:					
GLSM	3.70	63.80	62.90	5.06	0.83
SD	1.72	17.60	16.90	2.77	0.41
Minimum value	1	10	10	3	0.20
Maximum value	10.50	95	88	44	3.80
Coefficient of variation (CV %)	46	28	27	55	50
Heritability estimates and proportion of permanent environmental effects:					
h^2^ ± SE	0.17 (0.05)	0.28 (0.08)	0.27 (0.07)	0.27 (0.09)	0.23 (0.07)
* p*^*2*^ ± SE	0.16 (0.03)	0.37 (0.06)	0.35 (0.05)	0.43 (0.07)	0.46 (0.07)
e^2^ ± SE	0.67 (0.03)	0.35 (0.04)	0.38 (0.04)	0.30 (0.04)	0.31 (0.02)

Total number of records = 5178; EV = ejaculate volume, SM = Sperms Motility, LS = Live Sperms and AS = Abnormal Sperms and SC = Sperms Concentration

^**+**^GLSM = Generalized least square means estimated by Animal Model using PEST software, SD = standard deviations, SE = Stander error

## Estimated breeding values (EBVs) for semen traits

Estimates of minimum and maximum EBVs and their accuracy of predictions (${\text{r}}_{\text{A}}$) for semen traits are given in Table (4). Wide ranges of the EBVs were observed, being −0.69 to 1.27 ml for EV, −18.19 to 11.59% for SM, −19.31 to 9.15% for LS, −2.05 to 6.41% for AS and −0.39 to 0.54 × 10^9^ sperms per *ml* for SC. The percentages of positive EBVs for bulls with records and sires and dams of bulls without records for semen traits were medium, ranging from 38.05 to 61.06% (Table 4). The accuracies (r_A_) of minimum and maximum EBVs for semen traits were high, ranging from 0.55 to 0.97 (Table 4).

**Table 4:** Minimum and maximum estimated breeding values (EBV), their standard errors (SE) and accuracy of predictions (r_A_) for semen traits in Egyptian buffalo estimated by Single-trait Animal Model using BLUPF90 software

Trait	Minimum EBV	SE	r_A_	Maximum EBV	SE	r_A_	Range in EBV	Positive EBV (%)
EV (*ml*)	−0.69	0.14	0.71	1.27	0.41	0.97	1.96	46.90
SM (%)	−18.19	4.10	0.80	11.59	5.15	0.88	29.78	53.10
LS (%)	−19.31	3.93	0.79	9.15	4.94	0.87	28.46	53.21
AS (%)	−2.05	0.98	0.77	6.41	1.21	0.85	8.46	38.05
SC (10^9^ sperms per *ml*)	−0.39	0.21	0.55	0.54	0.28	0.89	0.93	61.06

Number of animals used = 237

## Genetic and phenotypic trends for semen traits

**Fig. 1:**
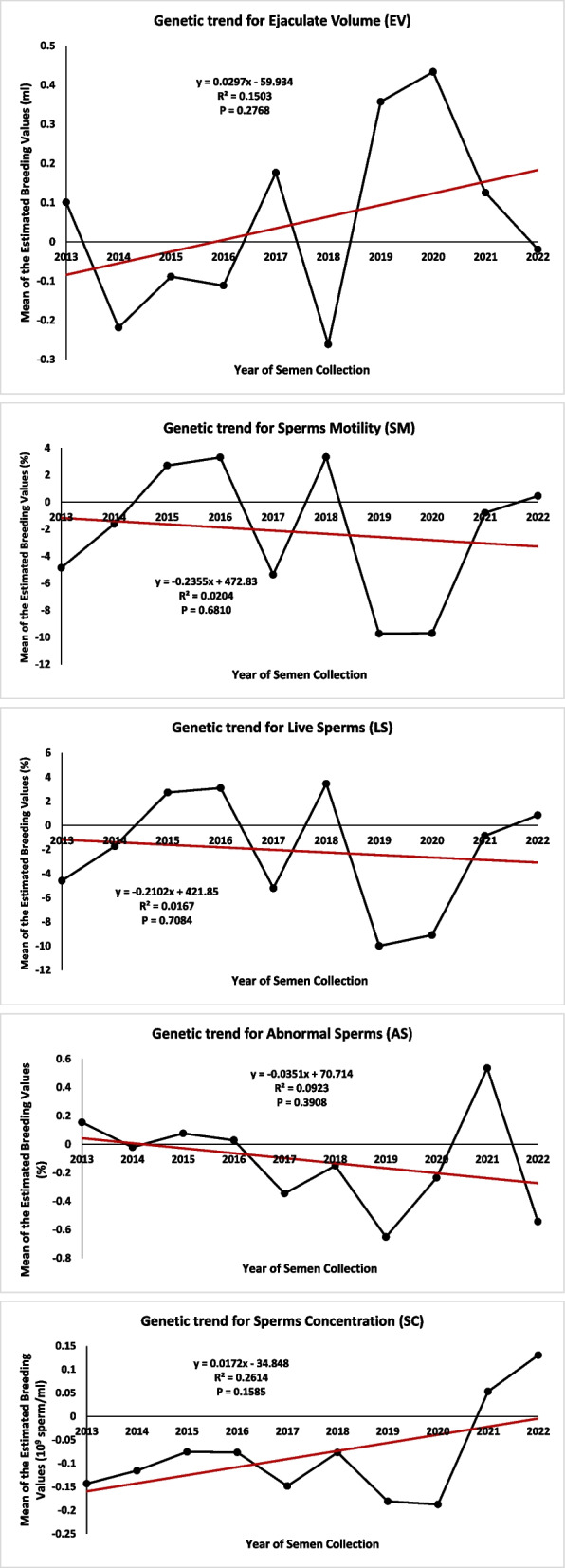
Genetic trends for semen traits in Egyptian buffalo. The trends plotted by regressing the annual means of the breeding values of semen trait on year of semen collection. Ejaculate Volume (EV), Sperms Motility (SM), Live Sperms (LS), Abnormal Sperms (AS) and Sperms Concentration (SC)

**Fig. 2:**
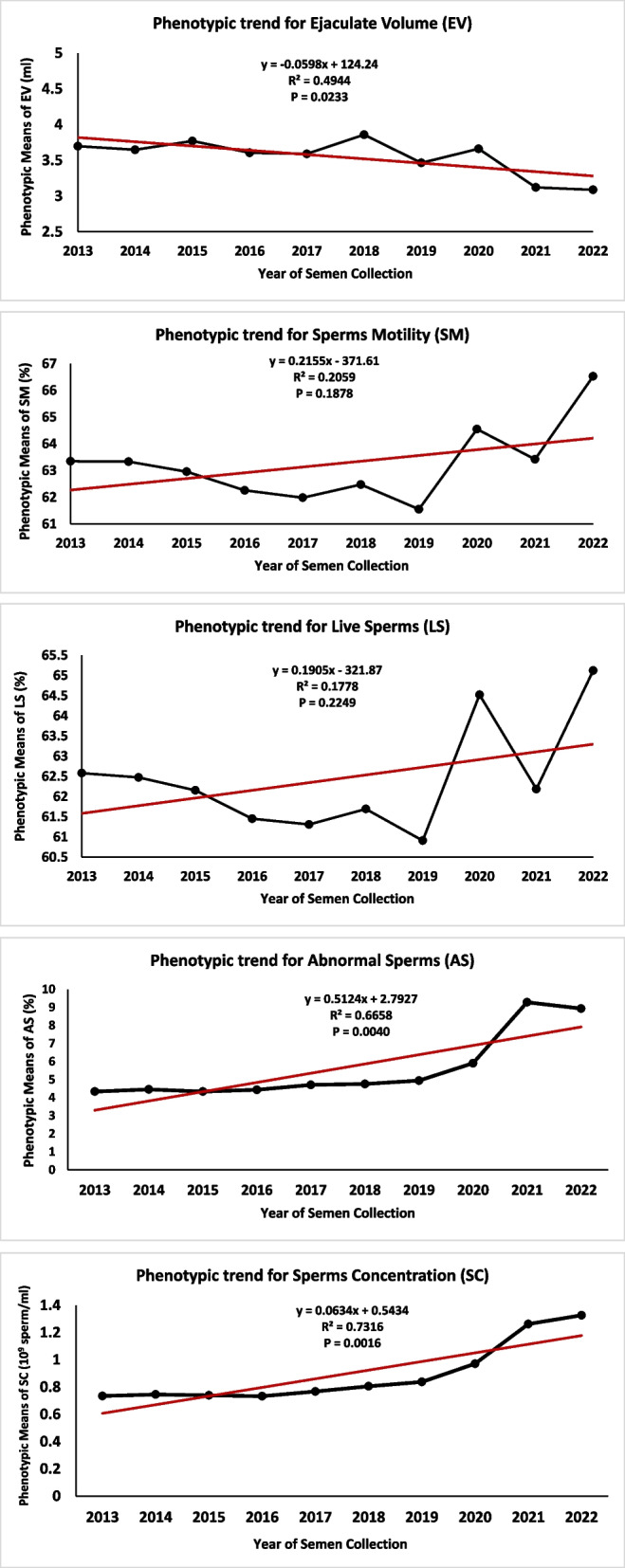
Phenotypic trends for semen traits in Egyptian buffalo. The trends plotted by regressing the GLSM of phenotypic values of semen traits on year of semen collection. Ejaculate Volume (EV), Sperms Motility (SM), Live Sperms (LS), Abnormal Sperms (AS) and Sperms Concentration (SC)

The genetic trends plotted for semen traits during the years from 2013 to 2022 are shown in Fig. 1. However, the genetic trends for all semen traits were insignificant, the genetic trend was favorably increased for EV and SC traits and decreased for SM, LS and AS traits. The averages of EBV throughout different years of semen collection were ranged from −0.26 to 0.43 ml for EV, −9.73 and 3.32% for SM, −9.99 and 3.45% for LS, −0.65 to 0.53% for AS and −0.19 to 0.13 × 10^9^ sperms per *ml* for SC. The phenotypic trends plotted for semen traits during the years from 2013 to 2022 are shown in Fig. 2. Although, R^2^ estimated for semen traits were relatively high in case of phenotypic trends compared to genetic trends, the phenotypic trends for EV, AS and SC traits were significant. The phenotypic trends plotted throughout the experimental period increased for all semen traits except for EV. The GLSM of semen phenotypic values throughout different years of semen collection were ranged from 3.09 to 3.86 ml for EV, 61.55 and 66.53% for SM, 60.91 and 65.12% for LS, 4.34 to 9.28% for AS and 0.73 to 1.33 × 10^9^ sperms per *ml* for SC.

## Molecular genetic associations between *FSHR* gene or *GH* gene and semen traits

The differences in GLSM for semen traits among GG, GC and CC genotypes of *FSHR* gene were significantly in favor of GG genotype (P < 0.01, Table 5). The differences in GLSM among GG, GC and CC genotypes of *FSHR* gene were significantly of 2.9 ml* vs* 2.6 and 2.5 ml for EV trait, 64.1*% vs* 59.3 and 63.2*%* for SM trait, 62.8*% vs* 57.8 and 61.9*%* for LS trait, 9.1*% vs* 9.9 and 9.4*%* for AS trait, 1.59 × 10^9^ sperms per *ml vs* 1.36 and 1.50 × 10^9^ sperms per *ml* for SC trait).

**Table 5:** Molecular associations between *FSHR* gene or *GH* gene and semen traits in the Egyptian buffalo expressed as generalized least square means and their standard errors (GLSM ± SE) estimated by PEST software

Semen traits	*FSHR* gene						*GH* gene			
	GG (N = 21)		GC (N = 20)		CC (N = 31)		TC (N = 50)		CC (N = 21)	
	GLSM	SE	GLSM	SE	GLSM	SE	GLSM	SE	GLSM	SE
Number of ejaculates	*N* = 189		*N* = 245		*N* = 279		*N* = 450		N = 188	
EV (ml)	2.9^a^	0.08	2.6^b^	0.08	2.5^b^	0.07	2.5^b^	0.06	2.9^a^	0.04
SM (%)	64.1^a^	0.91	59.3^c^	0.75	63.2^b^	0.81	60.9^b^	0.58	64.1^a^	0.92
LS (%)	62.8^a^	0.90	57.8^c^	0.74	61.9^b^	0.79	60.0^b^	0.58	62.1^a^	0.91
AS (%)	9.1^c^	0.29	9.9^a^	0.24	9.4^b^	0.25	9.6^a^	0.18	8.9^b^	0.28
SC (10^9^ sperms per *ml*)	1.59^a^	0.04	1.36^c^	0.34	1.50^b^	0.32	1.4^b^	0.03	1.6^a^	0.04

N = Number of semen records; EV = Ejaculate Volume; SM = Sperms Motility; LS = Live Sperms; AS = Abnormal Sperms; SC = Sperms Concentration

^a,^^b^ GLSM within each classification, not followed by the same letter in the row differed significantly (P < 0.01)

The molecular association analyses for semen traits revealed that two genotypes of CC and TC were detected or *GH* gene (Table 5). The associations were significantly in favor of CC genotype relative to TC genotype (P < 0.01). The GLSM for semen traits were significantly in favor of CC genotype compared to TC genotype (2.9 ml* vs* 2.5 ml for EV trait; 64.1*% vs* 60.9*%* for SM trait; 62.1*% vs* 60.0*%* for LS trait; 8.9*% vs* 9.6*%* for AS trait; 1.60 × 10^9^ sperms per *ml vs* 1.40 × 10^9^ sperms per *ml* for SC trait).

## Discussion

The studies concerning genetic analysis for semen traits in cattle and buffalo are scarce. Therefore, the genetic and phenotypic trends for semen traits in Egyptian buffalo, as well as their molecular genetic associations with *FSHR* and *GH* candidate genes, were first thoroughly outlined in the present study. The study seeks to provide insights into their potential role in improving fertility and reproductive efficiency in Egyptian buffalo breeding programs.

The GLSM for semen traits were in accordance with those means previously reported by several Egyptian investigators for Egyptian buffalo [2, 3, 24–26]. In this regard, Kadoom et al. [24] reported 60.5% for LS and 16.7% for AS, while Rushdi et al. [25] specified 66.20% for SM trait and 15.15% for AS trait. Similarly, wide variation for semen traits in Egyptian buffalo were reported by Khattab et al. [3], being 38.61% for AS, 21.86% for LS and 26% for SM. Salem et al. [4] and Amin et al. [26], reported GLSM of 46.57% for EV, 25.17% for SM, 25.17% for LS, 43.53% for AS and 24.49% for SC in Egyptian buffalo. Moreover, El Basuini et al. [2] evaluating some semen traits in Egyptian buffalo, stated that the coefficients of variation were 38.7, 21.83 and 25.93% for EV, LS and total motility, respectively.

The present estimates of heritability for semen traits were moderate, *i.e.* selection for semen traits in Egyptian buffalo could be performed efficiently. In available Egyptian buffalo literature, the heritability for semen traits were mostly moderate and ranged from 0.08 to 0.40 for EV, 0.06 to 0.42 for SM, 0.09 to 0.41 for LS, 0.04 for AS and 0.46 to 0.49 for SC [2–4]. In accordance with the present results, El Basuini et al. [2] reported heritability estimates of 0.08, 0.27 and 0.24 for EV, LS and total motility traits in Egyptian buffalo. However, these estimates varied from one study to another and these differences in heritability for semen traits may be attributed to several factors such as the fixed effects and covariates considered in the model of analysis, structure of data used, genetic constitution of the buffalo type, and coefficients of inbreeding and the relationship coefficient matrix [4].

The moderate estimates of permanent environmental effects for semen traits in the current study agreed with other studies. In this respect, Salem et al. [4] showed that the proportions of permanent environmental effects for EV, SM, LS, AS and SC in Egyptian buffalo were low or moderate, being 0.06, 0.30, 0.29, 0.024 and 0.0.29, respectively. In Holstein dairy bulls, Mathevon et al. [27] reported that the permanent environmental effects were mostly low and ranged from 0.0 to 0.22 in bulls younger than 30 *mo* and ranged from 0.0 to 0.63 in mature bulls aged from 4 to 6 years old for ejaculate volume, sperms concentration, motility of spams and total sperms.

The high genetic variabilities in semen traits suggested that there are promising prospects for selecting Egyptian buffalo bulls to enhance semen traits. Similarly, the reviewed ranges of EBVs were −0.448 to 3.32 ml for EV, −4. 28 to 52% for SM, −5.85 to 8.10% for LS and 799 to 1959 million per ml for SC in Egyptian buffalo [28] and in Indian Murrah buffalo [5]. In cattle studies, the ranges of breeding values were −7.10 to 11.0 ml for EV, −16.97 to 11.62% for SM and −336 to 428 × 10^6^ sperm per *ml* for SC [29–31].

The high accuracies in EBVs may be due to the fact that heritabilities for semen traits were highly associated with more available pedigree information for buffalo bulls and their studied sires and dams [4]. However, high accuracies in EBVs obtained in the present study indicate that selection of the Egyptian buffalo bulls could be used in the next generations of the present herds, and this would lead to sustainable genetic improvement for semen traits in Egyptian buffalo [4].

The wide ranges in genetic trends reflected suitable methodology of culling and replacement process practiced in buffalo herds of the present study. However, both genetic and phenotypic trends were plotted using data resulted from the same model of analysis, regressing the annual means of EBVs and GLSM of the phenotypic values on year of semen collection. Most of the phenotypic trends for semen traits were significant and all the genetic trends were not significant. The insignificant genetic trends for semen traits obtained in the present study could be expected and may be attributed to the following reasons: the relatively shallow depth of the pedigree, the limited number of years encompassed in the study, particularly considering that the generation interval in buffalo exceeds six years, thereby necessitating an extended duration to observe the genetic change adequately. Furthermore, the limited number of sires having progeny bulls with recorded semen characteristics. Moreover, the studied populations of Egyptian buffalo are being selected to enhance milk production related traits and not for semen traits. Studies in buffalo [5] and cattle [29] have shown that genetic and phenotypic trends for semen traits were favorable and showing considerable increase in both trends. Olsen et al. [29] found that the genetic trends in EV, SM and SC traits were increased in Norwegian Red cattle. Kumar et al. [5] showed that genetic and phenotypic trends were positive and showing favorable increase in EV and SM traits in Indian Murrah buffalo. It is worth to note in the current study that SM and LS traits are closely similar in phenotypic and genetic trends. This may be due to the high genetic correlation previously observed between both traits [4].

Sallam et al. [16] reported significant association between *FSHR* gene and sperm motility in Egyptian buffalo. Sang et al. [32] reported significant association between *FSHR* gene and EV and SC in Chinese Holstein cattle. Also, Nikitkina et al. [33] showed that the associations between *FSHR* gene and semen quality traits were significant (*P* < 0.05) for EV and SC and non-significant for SM trait. Recently, Khan et al. [34] reported that *FSHR* and other genes such as *INHA*, *INHAB*, *TNP2* and *SPEF2* were detected to be involved with sperm structural integrity, cellular communication, and DNA repair, all of which are critical for spermatogenesis and sperm function. The relationship between *FSHR* gene and the enhancement in semen traits in dairy bulls was previously explained by Yang et al. [35] who reported that a mutation in the SNP in the five upstream regions of the bovine *FSHR* gene may have changed the transcription-factor binding sites, which in turn may have changed the expression of the *FSHR* gene by affecting spermatogenesis in the testis and changing gene expression in the Sertoli cells. Jointly, the favourable impacts on sperm concentration and ejaculate volume may be accounted by the favourable genetic association between these traits.

In Egyptian buffalo, Darwish et al. [8] found that there were significant positive associations (*P* < 0.05) of LV genotype for GH gene with EV and SM traits. In dairy and beef bulls, Lechniak et al. [36] indicated that variations in *GH* gene genotypes may have an impact on the pattern of sperm production in bulls. In Holstein bulls, Afshar et al. [37] showed also that there were significant associations between genotypes of *GH* gene and semen traits, since LL genotype was the lowest in EV trait, while VV genotype was the highest in LS and SC traits. Moreover, Pal et al. [38] with two genotypes for *GH* gene (LL and LV) in a crossbred between Holstein Friesian and local Indian Tharparkar cattle, reported that LL genotype was positively associated with sperms motility, live sperms count, acrosomal integrity, hypo-osmotic swelling test (HOST), and number of semen doses per collection.

## Conclusion

Semen traits could be adopted as selection criteria to improve reproductive performance in Egyptian buffalo bulls. Improving management, feeding schemes and using accurate estimates of predicted breeding values in the genetic improvement programs, should improve semen traits of bulls in Egyptian buffalo efficiently. Wide ranges of the estimated breeding values for semen traits conjointly with the moderate heritability estimates could encourage improving these traits through selection. Based on the significant molecular genetic associations detected between semen traits and *FSHR* or *GH* genes, it could be used as potential candidate markers in marker-assisted selection schemes, aiming to improve reproduction efficiency traits in Egyptian buffalo. Future research could expand on these findings by incorporating larger sample sizes, exploring additional gene regions, and integrating genomic selection approaches to optimize breeding outcomes.

## Data Availability

Data availability No datasets were generated or analyzed during the current study. All results of the data used were presented through the manuscript. The raw data is available on reasonable requests from the corresponding author.
